# FXYD5: Na^+^/K^+^-ATPase Regulator in Health and Disease

**DOI:** 10.3389/fcell.2016.00026

**Published:** 2016-03-30

**Authors:** Irina Lubarski Gotliv

**Affiliations:** Department of Biological Chemistry, Weizmann Institute of ScienceRehovot, Israel

**Keywords:** FXYD5, dysadherin, Na^+^/K^+^-ATPase, polarity, cell adhesion, cell junctions, glycosylation

## Abstract

FXYD5 (Dysadherin, RIC) is a single span type I membrane protein that plays multiple roles in regulation of cellular functions. It is expressed in a variety of epithelial tissues and acts as an auxiliary subunit of the Na^+^/K^+^-ATPase. During the past decade, a correlation between enhanced expression of FXYD5 and tumor progression has been established for various tumor types. In this review, current knowledge on FXYD5 is discussed, including experimental data on the functional effects of FXYD5 on the Na^+^/K^+^-ATPase. FXYD5 modulates cellular junctions, influences chemokine production, and affects cell adhesion. The accumulated data may provide a basis for understanding the molecular mechanisms underlying FXYD5 mediated phenotypes.

FXYD5 (Dysadherin, RIC) is a type I membrane protein, that belongs to FXYD family. In mammalian cells this family of proteins consists of seven members (FXYD1-7) that share the conserved F-X-Y-D motif in the trans-membrane domain. All family members are known to interact with Na^+^/K^+^-ATPase and affect its kinetic properties in a tissue-specific manner (for a review see Garty and Karlish, 2006). FXYD5 was identified as a cell surface molecule by a monoclonal antibody that was developed to selectively recognize cancerous but not normal cells. Due to its effect of reducing cell–cell adhesion in transfected PLC/PRF/5 liver cancer cells the protein was termed dysadherin (Ino et al., 2002). Dysadherin and FXYD5 are synonyms for the same protein, consisting of 178 amino acid residues.

FXYD5 is expressed in a variety of cell types. Its role has been well characterized in epithelial tissues, probably due to the high abundance of FXYD5 in these cells and its up-regulation in several carcinomas, which originated from epithelial cells. FXYD5 was particularly abundant in intestine, spleen, lung, and kidney, and to much less extent in muscle tissues (Lubarski et al., 2005, 2007). Additional studies also recognized FXYD5 in endothelial cells and lymphocytes (Ino et al., 2002). The physiological significance of FXYD5 expression in the lymphatic system remains to be determined. The muscle tissues are a composite of a variety of cell types, and there is no indication yet, if FXYD5 expression in this tissue is myocyte specific. For instance, FXYD5 expression could be compartmentalized to endothelial cells, which are a part of the vascular supply to muscle bundle.

## FXYD5: Structure, molecular weight variability, and post translational modifications

Unlike other family members, FXYD5 has an atypically long extracellular domain >140 amino acids, including a cleavable signal peptide. FXYD5 has a short intracellular C-terminal segment of only 15 amino acids (Lubarski et al., 2005). The calculated molecular mass of FXYD5 is ~20 kDa, and without signal peptide the predicted molecular mass is ~17 kDa. In a variety of normal mouse epithelial tissue, a ~24 kDa polypeptide was reported to bind to two different antibodies raised independently against the synthetic C-terminal peptide and the truncated N-terminal domain (Lubarski et al., 2005, 2007). A similar molecular mass was also found for HA-tagged recombinant protein translated from the mouse FXYD5 cDNA in several cell lines (Lubarski et al., 2011, 2014). In agreement, Miller and Davis demonstrated that mature human Flag-tagged FXYD5 cDNA transfected into HEK 293 or LA4 cells produced protein that migrated as an indistinct band of ~35 kDa, and in mouse as a 25 kDa band (Miller and Davis, 2008a). However, in several cancerous cell lines antibodies recognized natively expressed FXYD5 of different molecular sizes, ranging between 50 and 55 kDa (Ino et al., 2002; Shimamura et al., 2004). The N-terminal domain, has a high abundance of Ser, Thr, and Pro residues, that were reported to be extensively *O*-glycosylated, an observation that could explain the variability in the apparent molecular weight of the protein (Ino et al., 2002; Tsuiji et al., 2003). Nevertheless, other explanations cannot be excluded. For example, detergent-resistant multimers in membrane preparations of cells expressing a FXYD5-Flag construct have been observed (Miller and Davis, 2008b). A similar phenomenon has also been reported for other FXYD proteins (Garty and Karlish, 2006; Wypijewski et al., 2013). Thus, the general conclusion is that some *O*-glycosylation is always present, since the migration size of native and expressed FXYD5 in all of the reported expression systems is consistently higher than the predicted molecular weight, and the presence of sugar moieties was demonstrated by lectin binding to the 24 kDa protein (Lubarski et al., 2011). Thus, the 50–55 kDa molecular species could be either a dimeric form of FXYD5 or a highly glycosylated species that is prevalent mainly in cancerous cells. In either case this issue deserves further investigation.

As discussed below, whereas the FXYD5 trans-membrane segment acts as a kinetic modulator of Na^+^/K^+^-ATPase activity, neither the N nor C extra-membrane segments of FXYD5 have been characterized in terms of possible functional effects (Lubarski et al., 2007). The intracellular region of FXYD5 is considerably shorter than that of the other FXYD family members and consists of only 15 amino acids. Interestingly, our observations have shown that any alterations to the C-terminus, such as deletion or tagging, results in failure of the protein to express at the surface, suggesting that the C-terminus is essential for correct folding or plasma membrane trafficking or both functions. In spite of its short length the C-terminus is known to undergo various post-translational modifications. For example, Ser^163^, a conserved residue in all FXYD proteins, has been suggested as a target for kinase-mediated regulation of cell motility in an *in vitro* model of airway epithelia. The authors concluded that phosphorylation at Ser^163^ regulates the FXYD5/Na^+^/K^+^-ATPase interaction and that this interaction modulates cell migration across a wound in airway epithelial cells (Miller and Davis, 2008b).

FXYD5 has two cysteines in its intracellular region, Cys^168^ and Cys^170^. Neither residue is exclusive for FXYD5 and both are located in equivalent positions throughout the FXYD family. Although the equivalent cysteines in all FXYD proteins are predicted to undergo palmitoylation, this modification has been demonstrated experimentally only for FXYD1 and FXYD5 (Tulloch et al., 2011; Martin et al., 2012). Palmitoylation of FXYD1 was reported to be promoted by PKC phosphorylation and to be required for inhibition of the Na^+^/K^+^-ATPase activity (Tulloch et al., 2011). The functional role of FXYD5 palmitoylation, if any, has not yet been characterized. Nevertheless, since all FXYD members have at least one conserved cysteine residue, palmitoylation may be a general means of regulating the pump.

At least one splice variant has been identified for FXYD5 with a non-canonical GT/CC splice site (Lubarski et al., 2007). Due to elimination of the original stop codon this splice variant creates a longer transcript encoding 10 additional residues at the C-terminus. Splice variants have also been reported for FXYD2 and FXYD3 (Kuster et al., 2000; Bibert et al., 2006). In both cases, differential expression of the two variants was observed, and in the case of FXYD3 different functional effects were also noted (Bibert et al., 2006). The functional effect of FXYD5 splice variant has not yet been analyzed. Table 1 summarizes the published data of all FXYD5 modifications and mutations.

**Table 1:** **Summary of all published FXYD5 modifications and mutations**.

**Residues**	**Location**	**Modification**	**Function**	**Expression system**	**References**
1–21	N-terminus	Signal peptide	Plasma membrane targeting	*Xenopus* oocytes	Lubarski et al., 2005
22–145	N-terminus	*O*-Glycosylation	Up-regulated in cancer?	PLC/PRF/5	Tsuiji et al., 2003
S^163^	C-terminus	Phosphorylation	Reduce association with Na^+^/K^+^-ATPase	MDCK	Miller and Davis, 2008b
C^168^, C^170^	C-terminus	Palmitoylation	Not tested	T-cells	Martin et al., 2012
^179^AYRVINMKES^188^	C-terminus	Splice variant	Not tested	Dendritic cells	Lubarski et al., 2007
A^150^, I^160^,L^161^	Transmembrane	Mutation to G,M,A	Reduce association with Na^+^/K^+^-ATPase	*Xenopus* oocytes, HEK293	Lubarski et al., 2007
R^145^	N-terminus, membrane interphase	Mutation to G	Increase association with Na^+^/K^+^-ATPase	HEK293	Lubarski et al., 2014

## Functional interaction of FXYD5 with Na^+^/K^+^-ATPase

The specific interaction of FXYD5 with Na^+^/K^+^-ATPase was demonstrated by co-immunoprecipitation in several expression systems (Lubarski et al., 2005, 2007, 2011; Miller and Davis, 2008b). Similar to FXYD1 (Crambert et al., 2002), but unlike FXYD4 and FXYD2 (Garty et al., 2002), association between FXYD5 and the Na^+^/K^+^-ATPase was found efficient also in the absence of Rb^+^/ouabain that preserve the native pump structure. The stability of the αβ/FXYD5 complex in detergent solubilized membranes and its high efficiency of co-immunoprecipitation are determined by its trans-membrane domain, as demonstrated by structure-function studies of FXYD5/FXYD4 chimeras expressed in *Xenopus* oocytes (Lubarski et al., 2007). A series of experiments, using point mutations, identified three trans-membrane residues as particularly important for the FXYD5/Na^+^/K^+^-ATPase interaction (Lubarski et al., 2007). All three residues, Ala^150^, Ile^160^, and Leu^161^, are common to all FXYD proteins, except FXYD4, and their significance for association with the pump were described in earlier studies (Lindzen et al., 2003; Li et al., 2004, 2005). A residue unique to FXYD5, Arg^145^, is located at the membrane- extracellular interface. Mutation of Arg^145^ to Gly (Arg^145^Gly), a residue located at the equivalent position in most of the FXYD family members, significantly increases the stability of the FXYD5/Na^+^/K^+^-ATPase complex, as demonstrated by co-immunoprecipitation experiments in HEK293 cells (Lubarski et al., 2014). Due to the location of Arg^145^ at the membrane-extracellular interface, the charge may have a major effect on the position of FXYD5 within the plasma membrane.

One of the well characterized functional effects of FXYD5 is to increase the pump activity (*V*_max_), measured either as ouabain-blockable and K^+^-induced outward current, or as ouabain-inhibitable ^86^Rb^+^ uptake. Co-expression of FXYD5 together with the Na^+^/K^+^-ATPase in *Xenopus laevis* oocytes produces a more than two-fold increase in the *V*_max_, without affecting the *K*_0.5_ for external K^+^ (Lubarski et al., 2005, 2007). In a separate study using Madin-Darby canine kidney cells (MDCK), transfected with FXYD5, the effect on *V*_max_ was also demonstrated. In addition, FXYD5 was shown to elevate the apparent affinity for Na^+^ about two-fold, and decrease the apparent affinity for K^+^ by 60% (Miller and Davis, 2008a).

FXYD5-FXYD4 chimeras were used to study the structure-function relations of different domains, measured by Rb^86^ uptake in *Xenopus* oocytes. These indicated that the FXYD5 trans-membrane segment is involved in the effect to increase the pumping rate. Other parameters were not tested in these experiments (Lubarski et al., 2007). Since the plasma membrane expression of Na^+^/K^+^-ATPase, as quantified by surface biotinylation, was not altered by FXYD5 expression, it was concluded that FXYD5 elevates the turnover rate of the pump (Lubarski et al., 2011).

The effects of FXYD5 on kinetic parameters of the Na^+^/K^+^-ATPase are small, about two-fold, similar to those reported for other FXYD proteins (Lubarski et al., 2005; Garty and Karlish, 2006). However, they are likely to be physiologically significant. The physiological role of the kinetic effect of FXYD5 can be proposed on the basis of its observed localization in normal tissue and on the basis of phenotypic analysis of differential FXYD5 expression under pathophysiological conditions. Interestingly, in kidney the FXYD5 cellular expression pattern correlates with low abundance of Na^+^/K^+^-ATPase. FXYD5 is expressed in cortical collecting duct intercalated cells, which have almost undetectable levels of the pump (Lubarski et al., 2005). Therefore, in principle, a FXYD5-mediated increase in *V*_max_ could serve as a homeostatic mechanism that acts to moderate or restore increased intracellular Na^+^ or decreased intracellular K^+^ ion concentrations, without altering the number of pumps in the cell. FXYD5 was found to be up-regulated during several pathological events. Patients with spinal cord injury (SCI) exhibited down-regulation of Na^+^/K^+^-ATPase, while FXYD5 expression was significantly increased (Boon et al., 2012). In alveolar epithelial cells, during hypoxia, up-regulation of FXYD5 was demonstrated, while several studies reported down regulation in Na^+^/K^+^-ATPase, at the transcriptional level and a decrease in the number of pumps at the basolateral membrane (Planès et al., 1996; Clerici and Matthay, 2000; Wodopia et al., 2000; Dada et al., 2003; Igwe et al., 2009). Up-regulation in FXYD5 under these conditions may serve as a compensatory mechanism to overcome the decrease in Na^+^/K^+^-ATPase unit numbers, by increasing the pumping rate. A significant increase in FXYD5 was found also in the lungs and nasal epithelium of cystic fibrosis (CF) mice, at the level of both protein and mRNA, and this up-regulation was directly correlated with loss of Cystic fibrosis transmembrane conductance regulator (CFTR) function (Miller and Davis, 2008a). Increased FXYD5 expression observed in CF airway epithelia may contribute to the increased Na^+^/K^+^-ATPase activity, which in turn may add to the greater sodium reabsorption seen in CF. The regulatory mechanism of FXYD5 increase under these pathological conditions is unknown yet. Nevertheless, FXYD5 up-regulation was reported following addition of pro-inflammatory cytokines TNFα or IL-1β (Miller and Davis, 2008a). Since inflammation is a common outcome of the above disorders it is a logical starting point in the evaluation of pathways regulating FXYD5 expression.

While structural interaction between FXYD proteins and the pump have been partially resolved by the crystal structures (Kanai et al., 2013), the mechanistic details of FXYD5 functional effects are still to be determined. It is certain, however, that the kinetic effects detected *in vitro* are not the only outcome of the FXYD5/Na^+^/K^+^-ATPase association as discussed below.

## FXYD5 expression is up-regulated in various human tumors

FXYD5 has been identified as a cancer-associated protein. In a number of clinical studies it was shown that there is a statistically significant correlation between the FXYD5 abundance and the progression of malignancies, accompanied by poor outcome of patients with various cancers (for a review see Nam et al., 2007). Initial studies showed that FXYD5 overexpression was correlated with reduced cell–cell adhesiveness, giving rise to the name “dysadherin” (Ino et al., 2002). In support of this concept, it was found that the mouse FXYD5 gene was induced in NIH 3T3 fibroblasts, transformed by a variety of oncogenes, including *E2a-Pbx1, v-Ras, Neu*, and *v-Src* (Fu and Kamps, 1997). Hence, clues to the basis of this FXYD5 mechanism of action is of high interest.

Some evidence has suggested the link of FXYD5 to known cancer promoting signaling pathways. In some cancer cell lines and tumors FXYD5 overexpression has been correlated with down-regulation of E-cadherin (Nakanishi et al., 2004; Batistatou et al., 2005; Kyzas et al., 2006; Sato et al., 2013). It has long been recognized that E-cadherin, acting as the cell–cell adhesion receptor, has an important role in suppression of tumor progression (Schipper et al., 1991; Oka et al., 1993; Umbas et al., 1994). Therefore, it was proposed that FXYD5 might promote metastasis by down-regulating E-cadherin. However, FXYD5 expression was correlated with changes in cell morphology *in vitro*, and with promotion of metastasis *in vivo*, in cell lines and tumors that did not express any E-cadherin. These findings suggest that E-cadherin dependence is not a general phenomenon for all FXYD5 expressing tumor types (Shimamura, 2003; Shimada, 2004; Nishizawa et al., 2005; Tamura et al., 2005; Lubarski et al., 2011).

A study, using a global gene expression analysis approach, identified chemokine (C-C motif) ligand 2 (CCL2) as the transcript most effected by silencing FXYD5 in MDA-MB-231 breast cancer cells (Nam et al., 2006). Investigations in animal model systems have recognized CCL2 as a mediator of inflammation with pro-malignant activities. CCL2 was shown to promote angiogenesis and act directly on the tumor cells to increase their migratory and invasion-related properties (Soria and Ben-Baruch, 2008). Correlation of FXYD5 expression with CCL2 secretion was demonstrated in different cell lines (Nam et al., 2006; Schüler et al., 2012). Furthermore, the ability of FXYD5 to promote invasion of MDA MB231 cells *in vivo* was inhibited by suppression of CCL2 (Nam et al., 2006).

In addition, more global, molecular changes were found to be associated with FXYD5 overexpression in breast cancer model. It was reported that FXYD5 expression results in enhancement of NF-κB transcriptional activity (Nam et al., 2006). The presence of FXYD5 was also accompanied by increased activation of AKT, demonstrated by elevated abundance of phospho-AKT in breast cancer tumors. Inhibition of AKT activity suppressed the ability of FXYD5 to promote the activation of the NF-κB pathway. Therefore, it was suggested that NF-κB activation might be a downstream signaling of FXYD5-mediated AKT activation (Lee et al., 2012).

Due to its ability to control cell proliferation and to suppress apoptosis, the transcription factor NF-κB is broadly related to carcinogenesis. Furthermore, the NF-κB pathway has been associated with control of metastasis and angiogenesis (Bassères and Baldwin, 2006; Karin, 2006). AKT is a serine/threonine kinase that functions through its ability to phosphorylate a number of key pro-oncogenic targets that promote cell growth or inhibit apoptotic pathways (Hay, 2005; Manning and Cantley, 2007). The NF-κB transcription factor has been identified as a target of the AKT signaling pathway (LoPiccolo et al., 2007).

In MDA-MB-231 cells, several hundred genes were reported to be differentially expressed associated with FXYD5 silencing, as determined by global gene expression analysis (Nam et al., 2006). Since NF-κB is a transcription factor that is responsible for a broad spectrum of cellular activities, it is possible that some of the pathways described above are initiated as a consequence of FXYD5-mediated NF-κB activation. However, it is not clear how FXYD5 alters NF-κB or any of the affected proteins described above. Since all the reported signaling events are modified at the transcriptional level, it is also unclear whether the proposed mechanisms are primary or secondary consequences of FXYD5 expression. Furthermore, direct interaction with FXYD5 has not been established with any protein other than Na^+^/K^+^-ATPase.

## FXYD5 over expression alters epithelial morphology

**Figure 1:**
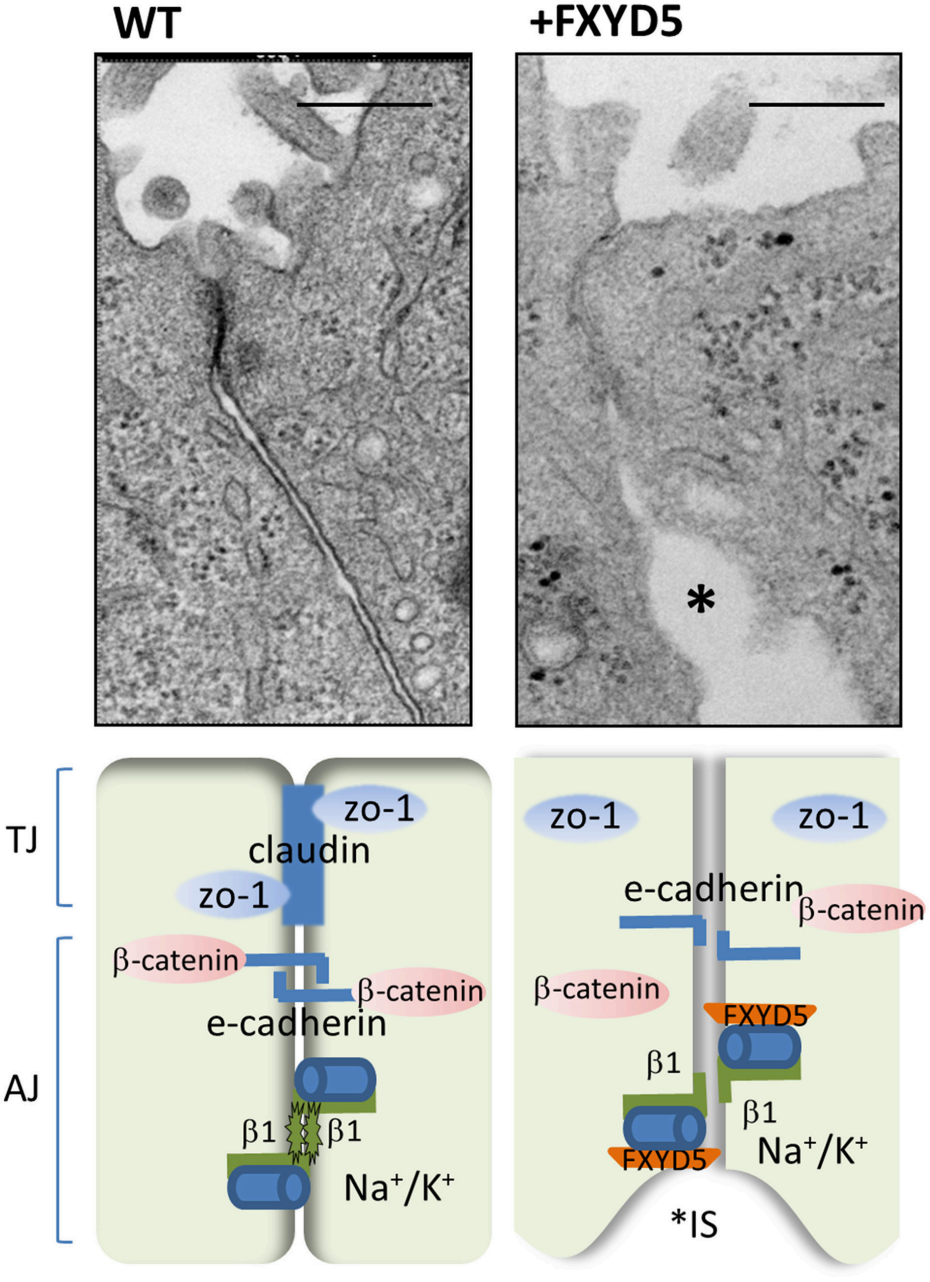
**FXYD5 impairs cell–cell junction formation**. (**Upper**) High magnification of electron microscopy images of normal and FXYD5 transfected M1 cell monolayer. Large expansions of interstitial spaces (IS) located just under the TJ evoked by FXYD5 expression (asterisks). Bar = 200 nm. The EM images were originally published in (Lubarski et al., 2011). (**Lower**) Experimental mode of FXYD5 function. FXYD5 expression in M1 polarized monolayer results in down regulation in membrane expression of TJ markers ZO-1 and occludin. E-cadherin membrane localization remained intact, while β-catenin, has been redistributed and appeared perpendicular to the membrane plane. A reduced form of β glycosylation has been observed, indicating an additional condition for promotion of “leaky” epithelia.

Since FXYD5 normally resides in kidney epithelia, it was logical to study the effects of its up-regulation in these cells. FXYD5 over expression was first examined in the rodent M1 cell, on account of their ability to create and maintain tight epithelial monolayer *in vitro*. In these cells, FXYD5 was shown to induce dilation of tight and adherent junctions (TJ, AJ) and promote expansion of interstitial spaces between the neighboring cells. These morphological changes were observed by electron microscopy and were further confirmed by re-distribution of TJ and AJ markers (ZO-1, occludin, and β-catenin) in FXYD5 expressing monolayers (Figure 1). As a consequence, FXYD5 transfected M1 cells lost their tight epithelial integrity, measured by reduced paracellular electrical resistance and increased trans-cellular permeability to macro molecules (Lubarski et al., 2011).

The effects of FXYD5 were also studied at a single cell level. It was found that the presence of FXYD5 results in impaired adhesion of cells to the matrix, as observed by the reduced rate of cell transformation from spherical to a flatter elongated shape, with inhibited anterior-posterior polarity. This morphological effect was seen consistently with several cell lines, and was demonstrated both by silencing FXYD5 in cells that normally express it (H1299, Panc-1), or transfecting FXYD5 into cells that lack this protein (M1, HEK293). Cells expressing FXYD5 exhibit a less flattened elongated shape, demonstrated also by a reduced number of focal adhesion points, as established by differential immuno-staining of paxillin, the focal adhesion- associated adaptor protein. Changes in focal adhesions were also correlated with less organized actin fibers structure (Lubarski et al., 2014).

Interestingly, there are some conflicting results concerning effects of FXYD5 on motility parameters in different cell lines. In some cell lines, the presence of FXYD5 evoked increased cell migration, in agreement with previously published data (Ino et al., 2002; Shimamura et al., 2004), whereas in others (M1 and H1299) the opposite results were obtained. This discrepancy was apparent for both single and collective cell movement. However, changes in FXYD5-mediated adhesion were constant and more robust finding that is not dependent on cell type (Lubarski et al., 2014).

Looser cell–cell contacts and impaired cell-matrix association could explain morphological changes associated with the role of FXYD5 in the metastatic processes. However, since FXYD5 is expressed in a variety of normal epithelia the effects described above might also be a component of its inherent physiological role. Distribution of FXYD5 in “leaky” epithelia of the gastro-intestinal tract could support this claim. An additional possibility is that FXYD5 is transiently up-regulated under conditions that require enhancement of paracellular permeability as, for example, during nutrient absorbtion or for infiltration of leukocytes during infection (Capaldo and Nusrat, 2009; Turner, 2009). The mechanism underlying additional functions of FXYD5 might also be explained as secondary effects following the increased pump activity. However, the phenotypes observed *in vitro* were apparent at very different pumping rates, arguing against this notion (Lubarski et al., 2011).

## FXYD5 additional effects might be mediated by its association with Na^+^/K^+^-ATPase

**Figure 2:**
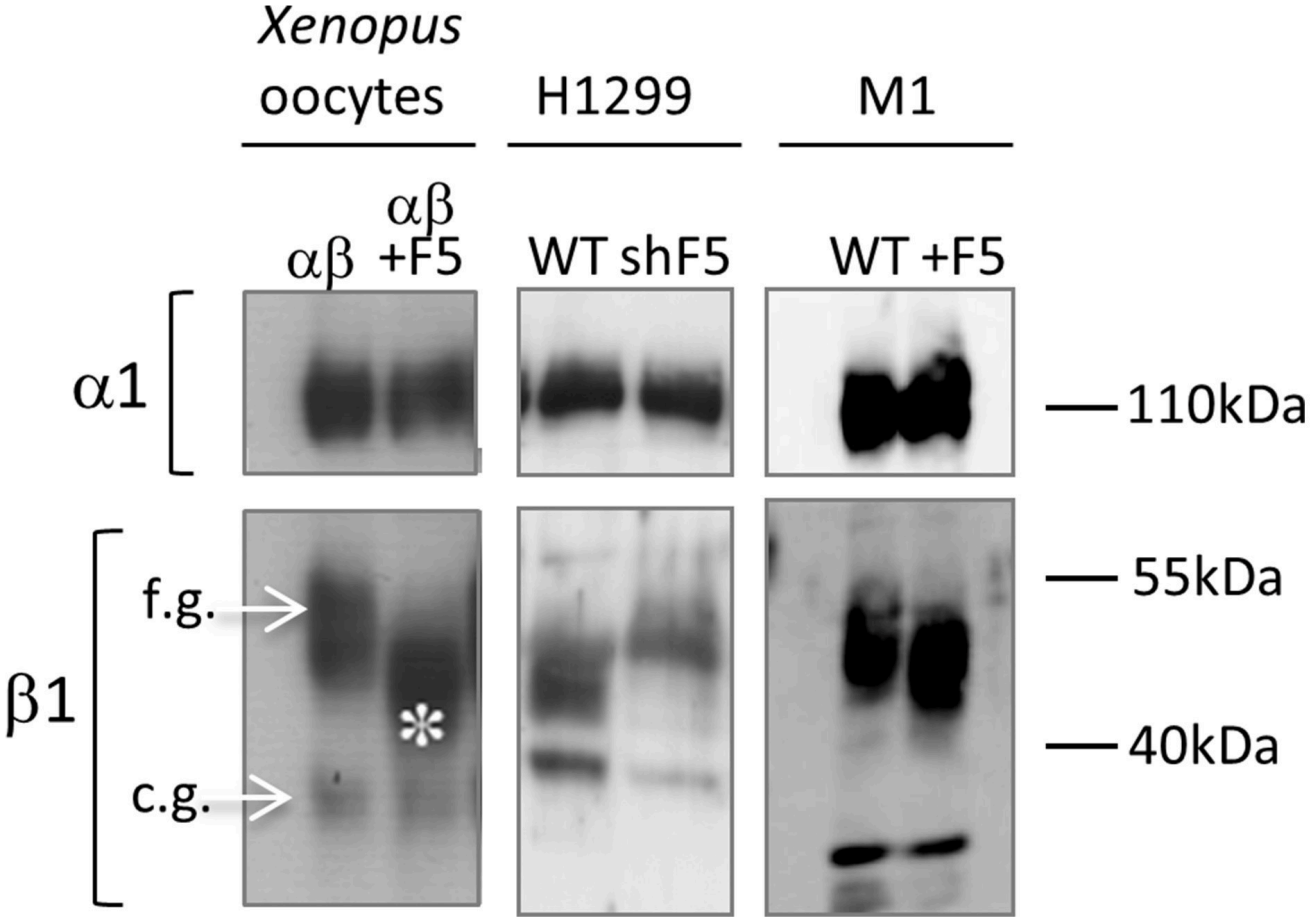
**Expression of FXYD5 effects β1-Na^+^/K^+^-ATPase glycosylation**. Western blot of microsomes: left panel- *Xenopus* oocytes, injected with αβ or αβ/FXYD5, middle panel- H1299 WT cells and shFXYD5 cells (shF5), right panel- M1 WT cell and FXYD5 transfected cells (+F5). β1-Na^+^/K^+^-ATPase is represented by two bands, one for fully glycosylated (f.g) and the other for core glycosylated (c.g.) form (marked with arrows). FXYD5 expression reduces glycosylation level of fully glycosylated form (asterisk). The figure was originally published in (Lubarski et al., 2007, 2011).

Another interesting effect of FXYD5 is the modification of the glycosylation state of the β1-subunit of Na^+^/K^+^-ATPase. The Na^+^/K^+^-ATPase pump consists of the catalytic α and structural β subunits. The classical role of β is to control assembly and stability of alpha-beta heterodimers and plasma membrane transport of sodium pumps (McDonough et al., 1990). The effect of FXYD5 on β1 glycosylation was noticed initially in *Xenopus* oocytes, and has been observed consistently with various mammalian expression systems that either normally express FXYD5 or have been transfected with FXYD5 cDNA (Figure 2; Lubarski et al., 2005, 2007, 2011). The phenomenon is manifested as increased migration of the highly glycosylated β1 form in SDS-PAGE gels, when co-expressed with FXYD5, with no change in its core unglycosylated protein. In general, this phenomenon may imply ER retention of an immature protein form. However, experiments determining the glycosylation state of surface biotinylated β1 seem to argue against such a possibility. Also, deglycosylation of β1 has no effect on the stability of the αβ complex, as reflected by surface expression of the α subunit in mammalian cells, and intact catalytic activity measured in *Xenopus* oocytes (Lubarski et al., 2005, 2011).

Various FXYD5/FXYD4 chimeras, constructed for structure-function studies, revealed that the presence of both trans-membrane and extracellular domains of FXYD5 are required for the reduced glycosylation of β1. Surprisingly, β deglycosylation is the only experimentally observed effect attributable to the long glycosylated extracellular portion of FXYD5, reported until now. Interestingly, a similar effect was demonstrated for FXYD3, but in this case the reduced glycosylation depended on the presence of uncleaved signal peptide (Crambert et al., 2005).

FXYD5-dependent modification of β1 glycosylation might be explained by a direct FXYD5-β interaction that may obstruct addition of sialic acid residues at all sites, or interfere with glycosylation at one or more positions. Although, according to the crystal structure (Toyoshima et al., 2011; Kanai et al., 2013) the transmembrane helices of the β and FXYD subunits are located at opposite sides of the α subunit, interactions between their extracellular domains cannot be ruled out. Nevertheless, if FXYD5-β interaction is possible, there is no certainty that it has an effect on β processing. At least in the case of FXYD3, it was demonstrated that the effect on glycosylation is not exclusive to β and furthermore, is not dependent on direct interaction with the Na^+^/K^+^-ATPase (Crambert et al., 2005). This evidence could indicate that FXYD5- mediated modification of glycosylation is not a direct effect, and might involve more global changes in protein sorting or in glycosylation machinery, or alternatively, that FXYD5 interacts with membrane proteins other than the Na^+^/K^+^-ATPase.

The functional significance of the above behavior may explain additional functions attributed to the β1 subunit. For example, several studies have provided evidence that the β1 subunit of Na^+^/K^+^-ATPase has a role as an adhesion molecule. Transcellular β1–β1 interactions have been shown to be involved in preservation and regulation of epithelial junctions, and the sugars moieties of the β extracellular domain were found to be important for maintaining β mediated cell–cell contacts (for a review see Vagin et al., 2012). Hence, FXYD5-mediated interference in β glycan structure, either direct or indirect, may result in “looser” junctions, characteristic of FXYD5 expressing cells.

The effect of FXYD5 on cell-matrix adhesion was also found to be mediated through its interaction with Na^+^/K^+^-ATPase. Specifically, two point mutations in the transmembrane segment demonstrated that association of FXYD5 with the pump is directly related with changes in cell morphology (Lubarski et al., 2014). Similar conclusions were drawn from Ser^163^Asp mutation. The negative charge at the mutated Ser^163^Asp residue, that mimics phosphorylation, has been proposed to regulate FXYD5/Na^+^/K^+^-ATPase association and this interaction has been correlated with modulation of collective cell movement in airway epithelial cells (Miller and Davis, 2008b). However, in contrast to the transmembrane mutations, the Ser^163^Asp mutation also interfered with plasma membrane localization of FXYD5.

## Key concepts and future research perspectives

FXYD5 is an established modulatory subunit of Na^+^/K^+^-ATPase, expressed in a variety of epithelial cells. Nevertheless, the function of its unique extracellular structural domain still remains elusive (Lubarski et al., 2005, 2007; Miller and Davis, 2008a). FXYD5 is up-regulated under several pathological conditions and its increased expression and differentially-modified molecular form in various malignancies pointed to possible functions in addition to kinetic modulation of Na^+^/K^+^-ATPase activity (Ino et al., 2002; Nam et al., 2007; Igwe et al., 2009; Boon et al., 2012). FXYD5-mediated alterations in cell morphology *in vitro*, and substantial transcriptional modification cannot be explained only by changes in Na^+^/K^+^-ATPase activity. The proposed molecular mechanisms underlying its effects in adhesion, motility, paracellular permeability, and metastatic progression vary between different cell types. The only consistently observed interaction of FXYD5 is with Na^+^/K^+^-ATPase. In addition to increasing the Na^+^/K^+^-ATPase pumping rate, FXYD5 has been shown to modify the glycosylation state of the β1 subunit (Lubarski et al., 2005, 2007, 2011). In parallel, a role of Na^+^/K^+^-ATPase as an adhesion molecule and the contribution of the β1 subunit to adherent junction formation have been established (Vagin et al., 2012). Therefore, a correlation between these processes has been proposed.

For the future a major focus should be on the physiological relevance underlying the effect of FXYD5 on Na^+^/K^+^-ATPase activity. The nature of FXYD5-mediated β1 carbohydrate modifications is a major question. This could call for a search for more global changes, since it appears obvious, that glycosylation of more than one protein is affected by FXYD5. In support of this notion, a role of another FXYD protein that shares this feature, FXYD3, has also been suspected in carcinogenesis (Maxwell et al., 2003). Altered glycosylation is a universal feature of cancer cells and it may explain several of FXYD5-induced morphological changes (Varki et al., 2009).

Finally, a most intriguing line of research could be to search for the trigger that activates transformation or modification of FXYD5 from its role in normal tissue to that in the malignant state. The reported up-regulation of FXYD5 in pathological conditions that require stabilization of the pump activity may shed light on the initial event that elicits malignant transformation.

## Author contributions

IL approved final version of manuscript, prepared figures, drafted manuscript, edited, and revised manuscript.

## Funding

Work in the author's laboratory was funded by research grants from the Israel Science Foundation and the Minerva Joseph Cohen Center.

### Conflict of interest statement

The author declares that the research was conducted in the absence of any commercial or financial relationships that could be construed as a potential conflict of interest.
